# Production of Hydroxy Fatty Acids, Precursors of γ-Hexalactone, Contributes to the Characteristic Sweet Aroma of Beef

**DOI:** 10.3390/metabo12040332

**Published:** 2022-04-06

**Authors:** Shuji Ueda, Mana Hosoda, Kumi Kasamatsu, Masahiro Horiuchi, Rio Nakabayashi, Bubwoong Kang, Masakazu Shinohara, Hiroki Nakanishi, Takayo Ohto-Nakanishi, Minoru Yamanoue, Yasuhito Shirai

**Affiliations:** 1 Department of Agrobioscience, Graduate School of Agricultural Science, Kobe University, Kobe 657-8501, Japan; 203a422a@stu.kobe-u.ac.jp (M.H.); kangb@pegasus.kobe-u.ac.jp (B.K.); yamanoue@kobe-u.ac.jp (M.Y.); shirai@kobe-u.ac.jp (Y.S.); 2 Research & Development, Takata Koryo Co., Ltd., Amagasaki 661-0001, Japan; k.kasamatsu@takatakoryo.co.jp (K.K.); m.horiuchi@takatakoryo.co.jp (M.H.); 3 Food Oil and Fat Research Laboratory, Miyoshi Oil & Fat Co., Ltd., Tokyo 124-8510, Japan; nakabayashir@miyoshi-yushi.co.jp; 4 The Integrated Center for Mass Spectrometry, Graduate School of Medicine, Kobe University, Kobe 650-0017, Japan; mashino@med.kobe-u.ac.jp; 5 Lipidome Lab Co., Ltd., Akita 010-0825, Japan; hnakani@lipidome.jp (H.N.); ohtakayo@lipidome.jp (T.O.-N.)

**Keywords:** Japanese black cattle, wagyu, fatty acids, lactone, quality, lipidomics, ALOX15, COX

## Abstract

Aroma is an essential factor for meat quality. The meat of Japanese Black cattle exhibits fine marbling and a rich and sweet aroma with a characteristic lactone composition. The mechanism of lactone formation associated with beef aroma has not been elucidated. In this study, we examined the precursors of γ-hexalactone, an indicator of the sweet aroma of beef and identified the mechanism underlying γ-hexalactone production. A low-temperature vacuum system was used to prepare beef tallow from Japanese Black cattle and Holstein cattle. The odor components were identified using headspace–gas chromatography. The analysis revealed that γ-hexalactone, γ-dodecalactone, δ-tetradecalactone, and δ-hexadecalactone were present as sweet aroma components of beef tallow prepared from marbling and muscle. Since we previously reported that γ-hexalactone formation correlates with linoleic acid content in beef, we analyzed ten oxidized fatty acids derived from linoleic acid by liquid chromatography–triple quadrupole mass spectrometry and detected two hydroxy-octadecadienoic acids (9S-HODE and 13S-HODE) in beef tallow. Significant differences in arachidonic acid 15-lipoxygenase and cyclooxygenase protein expression levels among subcutaneous fat, intramuscular fat, and muscle tissue were observed. Our results suggest that the combination of linoleic acid and the expression of lipid oxidase derived from beef muscle and intramuscular fat produce hydroxy fatty acids that result in a sweet aroma.

## 1. Introduction

In recent years, there has been a growing interest in sustainable food production. From the perspective of reducing the generation of greenhouse gases from livestock production [1], there have been increased efforts to develop plant-based or cell-based meats [2] as meat analogs [3]. The main issue in developing meat analogs is reproducing the appearance, flavor, palatability, and nutrition of sustainable resources [4].

Meat quality is strongly correlated with flavor and aroma. Beef aroma results from a complex mixture of hundreds of volatile compounds [5,6]. Organic acids, fatty acids, and sugars undergo Maillard reactions and pyrolysis during boiling or roasting, generating various volatile compounds. These compounds pass through the tip of the nose or throat to interact with olfactory receptors in the nasal cavity, which are then recognized as odors.

Japanese Black cattle (known as Japanese Wagyu) are one of the most expensive sources of beef in the world [7]. The distinguishing feature of Japanese Black cattle is a fine marbling consisting of intramuscular fat, which is based on genetic background [8,9,10]. Beef from Japanese Black cattle produces a rich and sweet aroma during thermal cooking [11,12]. This sweet aroma results from lactones of a cyclic ester and is known as the “Wagyu beef aroma” [13]. Holstein cattle are a breed of dairy cattle and are often used as a control for testing Japanese Black cattle, because they have lean muscle with little fat tissue. Previous studies revealed that the lactone composition produced after cooking differs between Japanese Black cattle and Holstein cattle [14]. Lactones are heterocyclic compounds that result from the dehydrating condensation of hydroxy groups (-OH) and carboxyl groups (-COOH) in the same molecule, such as a hydroxy fatty acid [15]. The sweet aroma of beef results from γ-lactones and δ-lactones with different heterocyclic rings. The γ-lactone has a nutty or sweet fruit aroma, whereas the δ-lactone has a coconut or butter-like aroma. As the number of carbons in the alkyl chain increases, these lactones change from a light, sweet aroma to a heavy, waxy aroma (over 14 carbons). The intensity and quality of the sweet aroma result from various types of lactones [12,13,14].

In a previous study, we identified γ-hexalactone as an indicator of the strength of the Wagyu beef aroma [14]. This γ-hexalactone is a unique lactone produced from marbled tissue rather than intermuscular fat, and the amount of γ-hexalactone correlates with linoleic acid [16]. Lactones are present in various food and drink products, such as beer [17], liquors [18], and ripening fruits [19]; however, the basis for the formation of γ-hexalactones in foods has not been elucidated. In particular, solid foods, such as beef, have not been well-studied compared with other foods because it is challenging to extract the components, such as the precursors for trace aroma. In beef aroma analysis, solid-phase microextraction (SPME) or solvent-assisted flavor evaporation (SAFE) methods have been developed to collect the odor components [11,12,13,20,21]. However, the marbling of Japanese Black cattle exhibits an uneven distribution in the muscle tissue and intramuscular fat, which causes difficulty in reproducibility and analysis using the SPME or SAFE methods.

This study focused on the lipid composition in beef tissue and the mechanism that generates the sweet aroma in beef. First, we extracted beef tallow from fat, marbled, and muscle tissue and analyzed the aroma components, such as lactones, that are produced during cooking. Next, we quantified the oxidized fatty acids derived from linoleic acid, the precursor of γ-hexalactone, as an indicator of the sweet aroma of beef in each tissue. Finally, we analyzed the expression of lipoxygenase and cyclooxygenase, which are involved in producing oxidized fatty acids, and linked the sweet aroma to beef tissue.

## 2. Results

### 2.1. Preparation of Beef Tallow by Vacuum Distillation

To explore the precursors of lactones in beef aroma, we divided beef samples into three tissues and extracted beef tallow. Low-temperature, high-speed vacuum distillation (#FED-50; water ejector vacuum dryer) was used to prevent the denaturation of lipids. The marbled tissue (called Marble) and fat tissue (Fats) of the longissimus thoracis of Japanese Black cattle, or muscle tissue (Muscle) of the longissimus thoracis of Holstein cattle were used for extracting beef tallow. We used lean muscle from Holstein cattle because the large amounts of lean muscle tissue from Japanese Black cattle cannot be separated from intramuscular fat. The water derived from the muscle plasma in the beef was removed as evaporated water under vacuum. Table 1 shows the percentages of beef tallow, dried solids, and evaporated water collected by vacuum distillation. The yield of beef tallow was highest in Fats (mainly intermuscular fat) at 56.8%, followed by Marble (muscle tissue and intramuscular fat) at 35.5%, and Muscle at 12.8% (Table 1). The amount of beef tallow prepared from the Muscle was only one-third for the Marble and one-fifth for the Fats.

**Figure 1:**
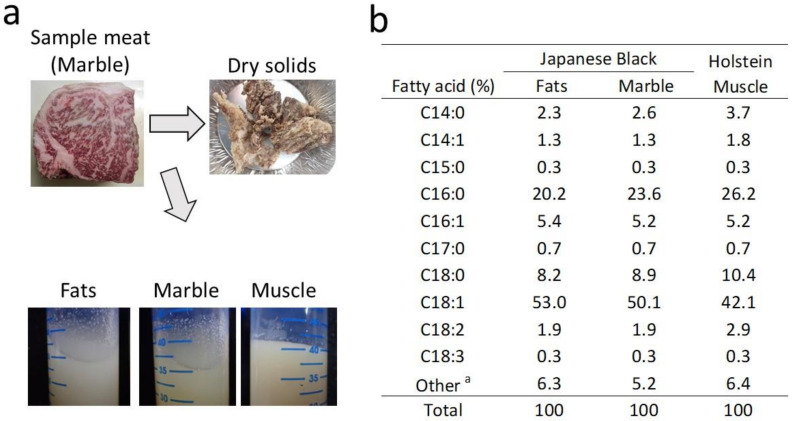
Comparison of low-temperature-vacuum-extracted beef tallow. (**a**) The images show the sample meat, beef tallow, and dry solids. The images above show the sample meat and dried solids derived from the marbling of the longissimus muscle of Japanese Black cattle. The images below show the surface condition of beef tallow after incubation at 30 °C for 1 h. (**b**) The table shows the proportion of fatty acid in beef tallow collected by vacuum distillation. ^a^ Other indicates the total peak area of the unidentified molecular species.

Japanese Black cattle beef contains more monounsaturated fatty acids compared with other cattle breeds [16]. In beef tallow, the Fats and Marble of Japanese Black cattle exhibited a lower melting point compared with the Muscle (Figure 1a). Dry solids (residues) ranged from 29.2% to 34.6% in the Fats, Marble, and Muscle. Muscle tissue tended to produce slightly more dry solids. The predominant components of the dry solids were connective tissue and muscle fibers.

In contrast, Muscle (52.7%) contained more evaporated water than Fats (14.1%) and Marble (31.2%) in Table 1. The table in Figure 1b shows the fatty acid composition of each beef tallow sample. The prepared beef tallow primarily consisted of lipids, and the fatty acid composition of the total lipid included oleic acid (C18:1), palmitic acid (C16:0), and stearic acid (C18:0). Oleic acid content was higher in the Fats and Marble of the Japanese Black cattle, whereas palmitic acid, stearic acid, and linoleic acid (C18:2) content was higher in the Holstein muscle tissue.

**Figure 2:**
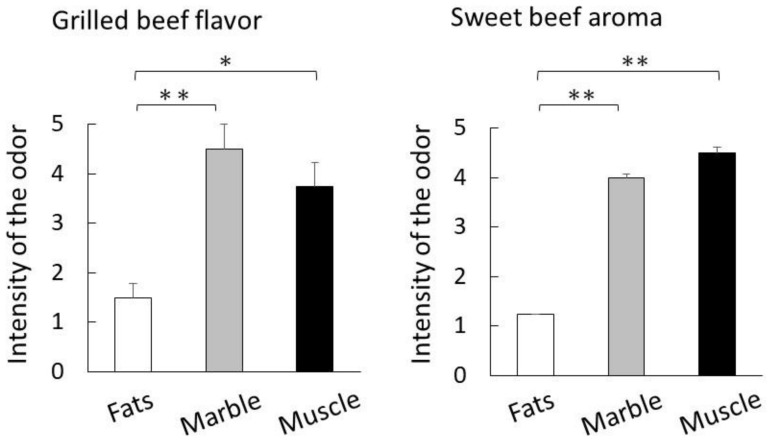
Aroma characteristics of prepared beef tallow by sensory evaluation. The bar graph shows the intensity of the beef tallow aroma after cooking (mean, four panelists). Significant differences are shown as follows. ** *p* < 0.01, * *p* < 0.05 (Tukey’s test; Fats vs. Marble vs. Muscle).

Beef tallow is also known to have a sweet aroma after cooking [14]. The results of a sensory evaluation of the prepared beef tallow indicate that the evaluation score for sweet aroma was highest for Muscle, followed by Marble, and then Fats (Figure 2).

### 2.2. Quantification of Odorants by Dynamic Headspace–Gas Chromatography–Mass Spectroscopy

**Figure 3:**
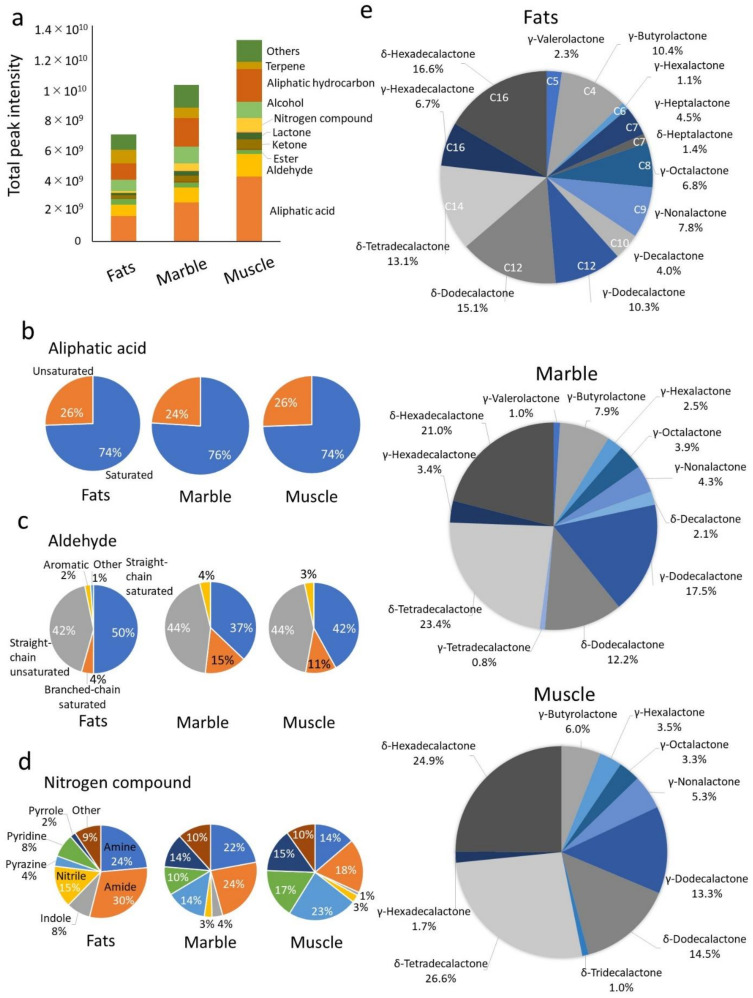
Analysis of aroma components generated from beef tallow by dynamic headspace–gas chromatography–mass spectroscopy. (**a**) Composition of the main functional groups of odorants in beef tallow. The bar graph shows the total intensity the summed intensities of detected compound peaks as classified by functional group. (**b**–**e**) Composition of lactone produced from beef tallow extracted from intermuscular fat of Japanese Black cattle. The pie chart shows the lactones produced by Fats, Marble, and Muscle. All graphs show the mean of the values obtained from three analyses.

The components of cooked beef aroma are complex. Thermal cooking produces hydrocarbons, aldehydes, ketones, carbonyl compounds, alcohols, and the cyclic compound, lactone, as odorants from beef [6,14]. A dynamic headspace system was used to analyze the odorants produced by thermal cooking from beef tallow. Fatty acids, aliphatic hydrocarbons, and aldehydes accounted for the majority of odorants in the beef tallow (Figure 3a). Marble and Muscle contributed more odorants than Fats. The ratio of esters and terpenes was higher in Fats compared with the other tissues, whereas lactones, ketones, aliphatic acid, aldehydes, and aliphatic hydrocarbon were higher in Marble and Muscle than in Fats. In particular, nitrogen compounds were significantly increased in Marble and Muscle.

We compared the ratios of the volatile components among beef tallow that exhibit the characteristic odors. Aliphatic acids, the most abundant in all beef tallow, are produced by heat oxidation of fatty acids [22]. For aliphatic acids, saturated aliphatic acids were identified in Fats (74%), Marble (76%), and Muscle (78%), and there was little difference in the ratio of saturated to unsaturated aliphatic acids in the beef tallow samples (Figure 3b). Figure S2 shows the composition of the saturated aliphatic acids. The aliphatic acids volatilized from beef tallow contained more than 14 carbons (70%), with tetradecanoic acid (C14; 39%), hexadecanoic acid (C16; 34%), dodecanoic acid (C12; 7%), and decanoic acid (C10; 5%) detected in Marble, in that order.

With respect to the aldehydes, the ratio of saturated aldehydes was higher compared with that of unsaturated aldehydes. Saturated and unsaturated aliphatic acids were detected at approximately the same ratio in all beef tallow samples. Figure S3 shows the composition of the saturated straight-chain aldehydes, which ranged from a low molecular weight to nonenal (C9) in length and differed in composition among the beef tallow samples. Fats tended to contain more propanal (C3; 4%) and nonenal (C9; 28%), whereas hexanal (C6; 44%) was detected in Muscle. Branched-chain saturated aldehydes were detected in abundance in Marble (15%) and Muscle (11%) as shown in Figure 3c. For nitrogen compounds, pyrrole was detected at a higher level in Marble (14%) and Muscle (23%) compared with Fats (Figure 3d). In contrast, the amide, indole, and nitrile ratios were higher in Fats.

Lactones are responsible for the sweet aroma of cooked Japanese Black cattle beef [12,16]. Figure 3e shows the composition of lactones produced from the beef tallow samples. High-molecular-weight lactones with more than ten carbons were present in abundance in beef tallow. γ-Haptalactone (C7), δ-heptalactone (C7), and γ-octalactone (C8) were detected at higher levels in Fats than in Marble and Muscle. In contrast, γ-dodecalactone (C12), δ-tetradecalactone (C14), and δ-hexadecalactone (C16) were more abundant in Marble and Muscle. γ-Hexalactone (C6), an indicator of the Wagyu beef aroma, was detected Fats (1.1%), Marble (2.5%), and Muscle (3.5%), in descending order.

### 2.3. Analysis of Hydroxylated Metabolites by Liquid Chromatography–Mass Spectrometry

**Figure 4:**
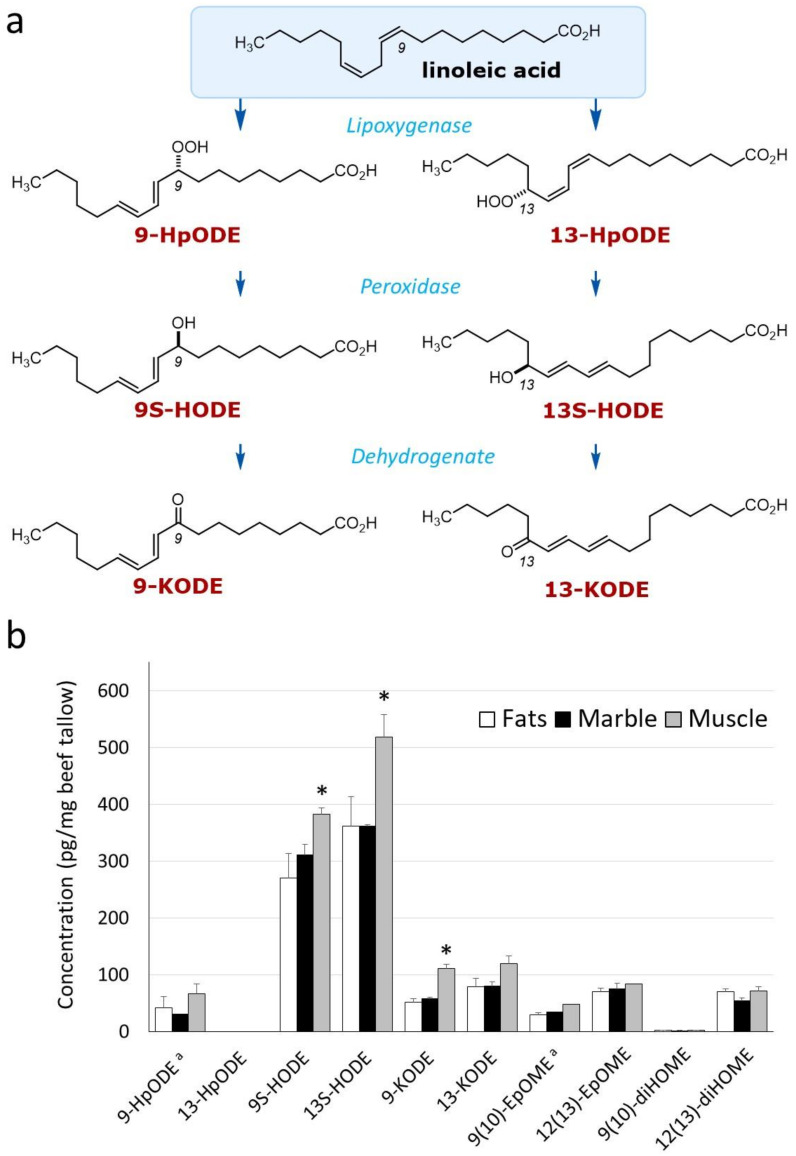
Quantitation of oxidative metabolites of linoleic acid. (**a**) Schematic diagram of lipid oxidation of linoleic acid. (**b**) Composition of oxidized linoleic acid in beef tallow. The graph shows the mean values obtained from three analyses of independently prepared samples. Data represent the mean ± standard error (*n* = 3). Significant differences are shown as follows. * *p* < 0.05 (Tukey’s test; Fats vs. Marble vs. Muscle). ^a^ indicates compounds below the lower limit of quantitation.

Lactones are formed during cyclization reactions of oxidized fatty acids [23]. γ-hexalactone was shown to correlate with the linoleic acid (c18:2 n-6) content of beef in our previous report [16]. We hypothesized that oxidized linoleic acid is a precursor to lactone formation and is responsible for the characteristic sweet aroma produced by thermal cooking. Linoleic acid contains two cis double bonds, and its chemical structure is susceptible to oxidation at various positions. The structure and oxidation pathway for linoleic acid are shown in Figure 4a. We used high-resolution liquid chromatography coupled with a triple quadrupole mass spectrometer system (LC-MS/MS) to identify ten oxidized fatty acids derived from linoleic acid. As oxidized fatty acids, hydroxy-octadecadienoic acids, 9S-HODE, and 13S-HODE were present predominantly in beef tallow (Figure 4b). Other oxidized fatty acids that exceeded the threshold for measurement were limited, except for oxo-octadecadienoic acids, 9-KODE, and 13-KODE. Both 9S-HODE and 13S-HODE were significantly higher in Muscle compared with the other beef tallow samples.

There are two types of reactions that oxidize linoleic acid: a free-radical reaction and an enzymatic reaction by oxidase. Oxidation occurs randomly in the free-radical reaction, whereas the enzymatic reaction is substrate-specific [24]. Comparison of 9S-HODE and 13S-HODE revealed a significant difference in beef tallow (Table 2), suggesting that an enzymatic reaction is involved in forming linoleic-acid-derived hydroxy fatty acids [25].

### 2.4. Expression Analysis of Linoleic-Acid-Related Oxidase in Muscle and Fat Tissues

**Figure 5:**
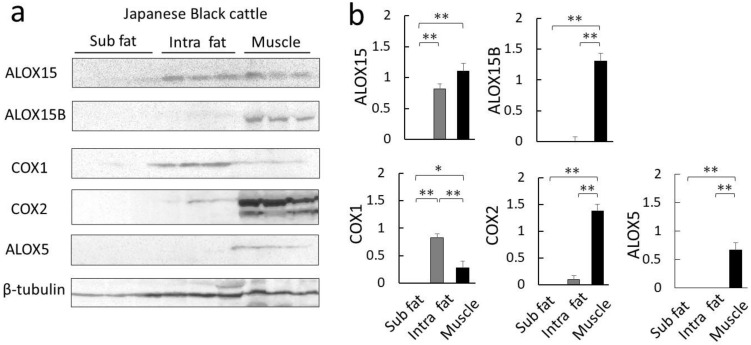
Comparison of lipid oxidase expression in Japanese Black cattle. (**a**) Western blot analysis using specific antibodies against lipoxygenase or cyclooxygenase was used to detect protein levels in subcutaneous fat (Sub fat), intramuscular fat (Int fat), and muscle tissue. The samples consisted of total proteins prepared from the sternocleidomastoid muscle of Japanese Black cattle. Samples prepared from three different cattle were analyzed. (**b**) The graph shows the mean of the intensity values detected by Western blot analysis. The data represent the mean ± standard error (*n* = 3). Significant differences are shown as follows: * *p* < 0.05 and ** *p* < 0.01 (Tukey’s test; Sub fat vs. Int fat vs. muscle tissue for each protein). Abbreviations: arachidonic acid 15-lipoxygenase (ALOX15), its isozyme ALOX15B, 5-lipoxygenase (ALOX5), cyclooxygenases 1 (COX1), and its isozyme COX2.

To search for enzymes involved in the production of lactone precursors [14], we examined the expression of linoleic-acid-related oxidase in Japanese Black cattle beef. In cattle, there are two isoforms of cyclooxygenase (COX) and six isoforms of arachidonic acid lipoxygenase (ALOX), which are enzymes that use linoleic acid as a substrate [8]. ALOX5, ALOX12, and ALOX15 hydroperoxylate 5, 12, or 15 carbons of arachidonic acid, respectively [26]. Five enzymes are candidates for the production of 9S-HODE and 13S-HODE: arachidonic acid 15-lipoxygenase (ALOX15) and its isozyme ALOX15B, as well as 5-lipoxygenase (ALOX5) [27], and cyclooxygenase 1 (COX1) and its isozyme COX2 [25,28]. We measured the expression of these enzymes in subcutaneous fat, intramuscular fat, and muscle tissue using Western blot analysis (Figure 5a). The results show that ALOX15B and COX2 were specifically expressed in muscle tissue (Figure 5a,b). In contrast, ALOX15 was highly expressed in both intramuscular fat and muscle tissue, whereas COX1 was abundantly expressed in intramuscular fat. None of the enzymes were detected in subcutaneous fat.

## 3. Discussion

In this study, we used a method to prepare beef tallow using a water ejector vacuum dryer (Figure 1). This equipment lowers the lipid melting point by low-temperature vacuum, enabling the extraction of beef tallow in a short period of time (Table 1). The advantage of this method is that it minimizes the Maillard reaction that occurs as a result of heating during conventional fat extraction [29]. Therefore, beef tallow prepared with this method retains more oxidized lipids and metabolites, which are precursors of the sweet aroma produced by thermal cooking. Supplementary Materials show the metabolites of beef tallow prepared from muscle tissue (Figure S1). The metabolites present in high amounts in beef tallow were glycerol, pyrophosphoric acid, and lactic acid. These metabolites were similar to the results of previous metabolomics analyses of adipose tissue from Japanese Black cattle [30]. No significant properties were observed in the major metabolites of beef tallow, regardless of tissue material. The evaporated water collected by low-temperature distillation exhibited a faint smell of some odor compounds, such as short-chain fatty acids and volatile organic acids. The evaporated water was not analyzed for any compounds in this study.

The fatty acid composition of the prepared beef tallow, which showed differences in oleic acid, palmitic acid, stearic acid, and linoleic acid, was consistent with that of a previous study [31]. Muscle had an approximately 1.5-fold higher percentage of linoleic acid compared with Fats and Marble (Figure 1b). Linoleic acid has been reported to be ten times more abundant in the phospholipids of cell membranes than in triglycerides [32]. This report suggests that muscle tissue with high cell density has abundant cell membranes, which may be why linoleic acid is more concentrated in Muscle than in Fats or Marble. In vitro studies with defatted meat have revealed that it is primarily phospholipids that produce the flavor of meat [5,33]. This difference in flavor between triacylglycerides and phospholipids appears to result from a higher content of unsaturated fatty acids in the phospholipids [34]. Determining the ratio of saturated to unsaturated fatty acids that produce the optimal meat flavor warrants further study.

In the sensory evaluation of the aroma of beef tallow, muscle-derived from Holstein muscle surprisingly scored the highest in the sweet aroma category (Figure 2). In previous evaluations, thermal-cooked Japanese Black cattle beef exhibited a sweeter aroma compared with Holstein beef [14]. This result suggests that even fat derived from Holstein cattle can produce a sweet aroma of Japanese Black cattle if the fat content is sufficient.

Analysis of beef tallow using dynamic headspace revealed a variety of odor components. Classification of these odorants revealed that the composition ratios of aliphatic acids, aliphatic hydrocarbons, aldehydes, and alcohols were similar among the beef tallow samples (Figure 3a). These odorants are produced by pyrolysis of lipid peroxides during the thermal oxidation of fatty acids. These aliphatic acids and aliphatic hydrocarbons constitute the odor of deep-fried oil [35] and are thought to form the backbone of the aroma generated from beef tallow. The fatty acids that are substrates for these reactions are presumably generated from the pyrolysis of triacylglycerides, which constitute the majority of beef tallow [36]. Myristic and palmitic acid produced by pyrolysis have been detected at a high frequency by gas chromatography–mass spectrometry in volatiles heated in the headspace (Figure S2). Interestingly, the volatile aliphatic acids produced by pyrolysis show little compositional difference in beef tallow. The composition of long-chain aliphatic acids (above C18) is unknown because of their low volatility, but it is thought that linoleic acid (C18:2) is also cleaved and remains in beef tallow.

We focused on aldehydes, nitrogen compounds, and lactones, which have low odor thresholds and are closely related to the aroma of beef tallow. Aldehydes are produced by lipid oxidation or the Strecker degradation of amino acids. These aldehydes represent the primary odorants in the flavor of grilled beef [6,37]. The aldehydes produced from beef tallow were more diverse compared with aliphatic acids (Figure 3b). In particular, Marble and Muscle exhibited high levels of branched-chain aldehydes (Figure 3c). These unique aldehydes are thought to be formed by components derived from muscle rather than adipose tissue. Nitrogen compounds contribute to the savory roasted flavor of beef [6]. The characteristic nitrogen compounds that were increased in Marble and Muscle were pyrazines (2,5-dimethylpyrazine and 2-ethyl-5-methylpyrazine) and pyrroles (2-acetylpyrrole). Dimethylpyrazine was detected as an odorant in roasted nuts [38], and 2,5-dimethylpyrazine is a notable flavoring agent involved in the favorable flavor of beef [39,40].

Lactones are the major sweet aroma component of the Wagyu beef flavor. We previously measured lactones produced in cooked beef using stable isotopes and found that Japanese Black cattle produced significantly more γ-lactones (C6, C7, C8, C9, C10) and δ-lactones (C10) than Holstein cattle [14]. For this analysis, the combination of vacuum-distilled beef tallow and dynamic headspace enabled the separation and detection of a wide range of lactones of varying molecular weight (Figure 3e). In the lactones generated from beef tallow, high-molecular-weight lactones (over 12 carbons), such as δ-tetradecalactone, δ-hexadecalactone, δ-dodecalactone, and γ-dodecalactone, accounted for more than 70% of the total detected lactones. This trend in high-molecular-weight lactone composition was also observed by Yoshinaga et al. [12], and a higher production of δ-tetradecalactone and δ-hexadecalactone was observed in lean meat compared with Japanese Black cattle. These high-molecular-weight lactones have a waxy aroma that masks odd odors [41]. They may be produced from stearic acid, which is more abundant in Muscle than in Fats and Marble (Figure 1b).

We focused on γ-hexalactone as an indicator of the strength of the Wagyu beef aroma and identified 9S-HODE and 13S-HODE as its precursor (Figure 4b). These oxidized fatty acids, which are derived from linoleic acid, function as lipid mediators in mammals. We hypothesized that HpODE, HODE, and KODE are intermediates in the pathway from linoleic acid to γ-hexalactone [24] as shown in Figure 4a. Using LC-MS/MS, 9-HpODE and 13-HpODE exhibited short half-lives and 9S-HODE and 13S-HODE were abundant in beef tallow (Figure 4b). HpODE is a hydroperoxide produced by lipoxygenase and reduced to HODE by peroxidase [24]. Compared with plant tissues, which are rich in polyunsaturated fatty acids [42,43], the amount of oxidized fatty acids in beef tallow was low. The ratio of 9S-HODE:13S-HODE varies among cells and tissues, which may indicate the stereospecificity of the oxidase [44].

We examined the expression of ALOX and COX, which are involved in the production of 9S-HODE and 13S-HODE. ALOX15 can lipid-oxidize linoleic acid and arachidonic acid as substrates to produce 13S-HODE [45]. On the other hand, ALOX15B, an ortholog of ALOX15, can lipid-oxidize like ALOX15 but does not produce 13S-HODE because it uses only arachidonic acid as a substrate [45]. Therefore, 13S-HODE is thought to be produced by linoleic acid oxidation by residual ALOX15 activity in intramuscular fat and muscle.

COX and ALOX5 preferentially contribute to the production of 9S-HODE from linoleic acid [24,25]. In our analysis, COX2 and ALOX5 expression were particularly prominent in muscle tissue (Figure 5). COX2 and ALOX5 are mainly involved in inflammatory responses, and in vivo, ALOX5 contributes to the formation of 5-hydroxyeicosatetraenoic acid (5-HETE) and COX2 to prostaglandin H using arachidonic acid as a substrate.

Obesity and some tumors activate COX2 and ALOX5 and induce inflammatory responses [46]. In addition, enhanced TGF-β signaling has been shown in the intramuscular fat of Japanese Black cattle [8]. It is suggested that the expression of COX and ALOX, which are involved in the production of these inflammatory mediators, may be induced in the intramuscular fat and muscle tissue of the Japanese Black cattle. The details of the regulation of expression, subcellular localization, and activity mechanisms of these lipid oxidase enzymes in the Japanese Black cattle are unknown.

**Figure 6:**
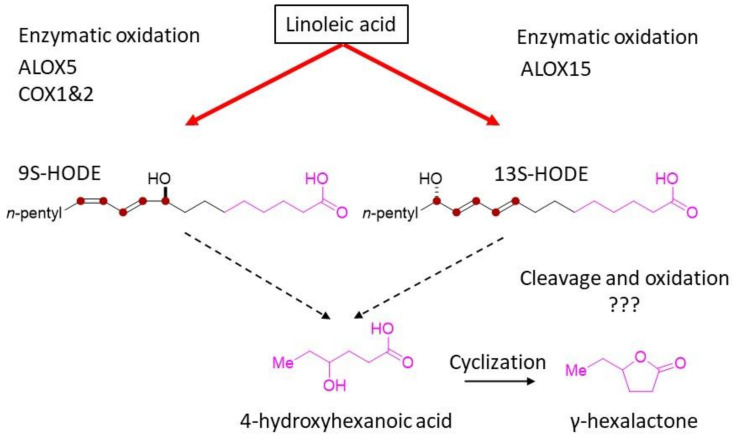
Proposed pathway of γ-hexalactone related to the sweet aroma of beef via enzymatic reaction. We propose a pathway for the formation of γ-hexalactones via intermediates 9S-HODE or 13S-HODE, which are produced from free linoleic acid in beef by enzymatic oxidation. These hydroxy fatty acids may be further degraded to 4-hydroxyhexanoic acid through cleavage and oxidation reactions, and then to γ-hexalactone by cyclization reactions. Question marks (???) indicates an unknown chemical reaction pathway.

These results suggest that the sweet aroma of beef may be related to hydroxy fatty acids produced by lipid-oxidizing enzymes in muscle and Marble when free unsaturated fatty acids supplied by adipose tissue meet sufficient conditions (Figure 6). How 13S-HODE and 9D-HODE are cleaved to 4-hydroxyhexanoic acid is an issue to be clarified in future studies.

## 4. Materials and Methods

### 4.1. Sample Collection

The intermuscular Fats and longissimus muscle of Japanese Black cattle (28–32 months of age, steer, total of 5 cattle, Japan) or longissimus muscle of Holstein cattle (18–19 months of age, steer, total of 5 cattle, Japan) were purchased commercially from livestock farmers and vacuum-packed at a meat processing center (Tokyo, Japan). The longissimus muscle was stored at 2 °C for 20 days after slaughter and frozen at −30 °C. All samples used in this study were collected during the process of commercial distribution of beef with the cooperation of ordinary livestock farmers, and no animal experiments were included.

### 4.2. Purification of Beef Tallow by Vacuum Extraction

Beef tallow was extracted using a water ejector vacuum dryer (#FED-50) manufactured by F.E.C. Corporation (Hyogo, Japan). The samples (6–9 kg) were applied to the drying kettle and separated from the beef into dried solids, aqueous solution, and extracted beef tallow by vacuum distillation (vacuum −98 kPa, temperature 37 ± 1 °C). The purified beef tallow was transferred to a sealed plastic container and stored frozen at −30 °C until analysis.

### 4.3. Analysis of Fatty Acids Composition

Total lipids were extracted from beef tallow (10 g) with t-butyl methyl ether–methanol (2:1), followed by purification using an InertSep SI column (GL Sciences, Tokyo, Japan). The fatty acid composition was analyzed by gas chromatography (GC-2010 Plus; Shimadzu, Kyoto, Japan) using a TC-70 capillary column (GL Sciences, Tokyo, Japan) under conditions described previously [16].

### 4.4. Analysis of Odor Components of Beef Tallow after Heating

A large dynamic headspace was used to collect odorants produced by heating beef tallow. Beef tallow (1.0 g) was heated in a dynamic headspace (DHS-Large 500 mL capacity; Gerstel K.K., Tokyo, Japan) at 200 °C (actual temperature 170–180 °C). Nitrogen gas was aerated at 50 mL/min for 5 min, and the odorants were trapped in a carbon-based adsorbent (carbopack B/carbopack X; Gerstel K.K., Tokyo, Japan) and dried by venting nitrogen gas at 25 mL/min for 10 min. The odorants were analyzed on a thermal desorption unit–gas chromatography–mass spectrometer (TDU-GC/MSD; Agilent Technologies Japan, Tokyo, Japan) using an Agilent 7890B gas chromatograph-5977B mass spectrometer equipped with a DB-WAX capillary column (length, 30 m; inner diameter, 0.32 mm; film thickness, 0.50 μm; Agilent Technologies, Tokyo, Japan). TDU was performed at 30–300 °C in low split mode. The oven temperature was maintained at 50 °C for 5 min and then at 220 °C for 60 min. The 5977B mass spectrometer was operated at a scan range of m/z 35–350, an ionization voltage of 70 eV, and an ion source temperature of 230 °C. Volatile compounds were identified by comparing their mass spectra and retention index to those of the TAKATA Koryo Co., Ltd., (Amagasaki, Japan) MS private library database developed using standard samples.

### 4.5. Sensory Evaluation

The aroma analysis of beef tallow was entrusted to the Japan Meat Science and Technology Research Institute (Tokyo, Japan) as reported in a previous paper [14]. Samples (1 g) were placed on aluminum foil, heated at 180 °C for 60 s using an electric griddle (Zojirushi Mahovin, Tokyo, Japan), and evaluated blindly for retronasal aroma by four professional analytical panelists.

### 4.6. LC-MS/MS Analysis of Hydroxy Fatty Acids

Total lipid fractions were extracted from purified beef tallow samples by single-step deproteinization using methanol. The oxidized fatty acid fraction was purified from the lipid fractions by solid-phase extraction with Oasis HLB columns (Waters Corporation, MA, USA). Hydroxy fatty acids were separated using a high-performance liquid chromatography system (Nexera LC-30AD, Shimadzu Corporation, Kyoto, Japan) equipped with an XBridge C18 column (particle size 3.5 µm, length 150 mm, inner diameter 1.0 mm; Waters) and analyzed on a triple quadrupole mass spectrometer (LC-MS-8040; Shimadzu, Kyoto, Japan).

Mass spectrometric analysis of hydroxy fatty acids was performed in negative ion mode with an injection volume of 15 µL containing 0.5 mg of the oxidized fatty acid fraction and 1500 pg of the internal standard (12,13-diHOME-d4, 13S-HODE-d4, 13-KODE-d3, 12,13-EpOME-d4; Cayman chemicals, Ann Arbor, Michigan USA). The quantification of hydroxy fatty acids was identified and quantified by multiple-reaction monitoring as reported for the determination of other lipid metabolites [47]. For quantitation, calibration curves were prepared for each compound, and recoveries were monitored using deuterated internal standards. Data analysis was performed using LabSolutions software (Shimadzu, Kyoto, Japan).

### 4.7. Western Blot Analysis

Subcutaneous fat, intramuscular fat, and muscle tissue were isolated from the sternocleidomastoid muscle of Japanese Black cattle (3 cattle, steers) [8]. Tissues (200 µg) were disrupted with a polytron homogenizer in RIPA buffer (25 mM Tris-HCl pH7.5, 150 mM NaCl, 1% Nonidet-P40, 1% sodium deoxycholate, 0.1% SDS) containing a protease inhibitor cocktail (Fujifilm Wako chemical, Osaka, Japan) as described above [48]. The supernatant was collected by centrifugation at 17,730× *g*. Samples of fat tissues were subjected to acetone precipitation using 10-fold volume of a cold acetone to remove excess lipids. After protein quantification by BCA protein assay kit (Fujifilm Wako chemical, Osaka, Japan), equal amounts of protein (22.5 µg/lane) were subjected to SDS-polyacrylamide gel electrophoresis and electrophoresed onto PVDF membranes (Immobilon-P; pore size 0.45 µm, Merck K.K., Tokyo, Japan).

After blocking with 5% skim milk in phosphate-buffered saline (PBS) with 0.05% Tween20, the membranes were incubated with specific primary antibodies diluted in PBS with 0.05% Tween20. The bound antibodies on the membrane were detected by chemiluminescence with a horseradish-peroxidase-conjugated secondary antibody (Jackson Immunoresearch Laboratories, West Grove, PA, USA) and ImmunoStar zeta detection reagents (Fujifilm Wako chemical, Osaka, Japan) on the Limited-STAGE (AMZ system science, Osaka, Japan). Signal intensity was measured with the NIH ImageJ software bundled with 64-bit Java 1.8.0_172 (https://imagej.nih.gov/ij/index.html, accessed on 5 September 2021). Primary antibodies were purchased from Cosmo Bio Inc (Tokyo, Japan). The details are as follows: anti-ALOX15 (#PAA891HU01, 1:250 dilution), anti-ALOX15B (#CSB-PA001622ESR2HU, 1:1000 dilution), anti-COX1 (#CSB-PA001761, 1:1000 dilution), anti-COX2 (#CSB-PA001764, 1:2000 dilution), and anti-ALOX5 (#WLS-PAB355HU01, 1:1000 dilution).

### 4.8. Statistical Analysis

Statistical significance was determined using a Student’s *t*-test with Excel 2019 (Microsoft Japan, Tokyo, Japan) or Tukey method with BellCurve (Social Survey Research Information, Tokyo, Japan).

## 5. Conclusions

In this study, we analyzed the mechanism of lactone production of the sweet aroma of Japanese Black cattle beef using beef tallow. Analysis of aroma components by headspace–gas chromatography detected numerous lactone components, including γ-hexalactone, γ-dodecalactone, δ-tetradecalactone, and δ-hexadecalactone. Since γ-hexalactone, an indicator of the Wagyu beef aroma, is correlated with linoleic acid, we assumed linoleic acid as a precursor and explored for oxidized fatty acids that are intermediates of γ-hexalactone using LC-MS/MS to identify 9S-HODE and 13S-HODE. Based on the formation ratios of these oxidized fatty acids, a specific enzymatic oxidation pathway, rather than random oxidation, was inferred. To further clarify the enzymatic reaction mechanism, we analyzed the expression of the relevant lipid oxidase enzymes, ALOX and COX, in each tissue of Japanese Black cattle. The results show that ALOX15B, ALOX5, and COX2 were highly expressed in muscle, while ALOX15 and COX1 were expressed in muscle and intramuscular fat. From the substrate specificity of lipid oxidase, 13S-HODE may involve ALOX15, and 9S-HODE may involve COX1, COX2, and ALOX5.

## Figures and Tables

**Table 1:** Low-temperature vacuum extraction of beef tallow using a water ejector vacuum dryer.

Sample Names	Tissue	Species	Vacuum Distillation (%)		
			Beef Tallow	Solid Content	Evaporated Water
Fats	Intermuscular fats	Japanese Black cattle	56.8	29.2	14.1
Marble	Muscle and intramuscular fats	Japanese Black cattle	35.5	33.3	31.2
Muscle	Muscle	Holstein cattle	12.8	34.6	52.7

**Table 2:** Low-temperature vacuum extraction of beef tallow using a water ejector vacuum dryer.

Metabolite	Japanese Black		Hostein Muscle
	Fats	Marble	
9-HpODE ^a^	42.0	30.7	66.7
13-HpODE	-	-	-
9-HODE	269.9	310.8	382.7
13-HODE	361.0	361.9 *	518.5 **
9-KODE	51.1	57.6	111.3
13-KODE	78.9	79.4	118.8
9-EpOME ^a^	29.1	33.6	47.6
12-EpOME	70.4	74.5	84.2
9-diHOME	1.83	1.43	2.21
12-diHOME	69.7	53.6	71.4
	Quantitative value (pg/mg beef tallow)		

The table shows the oxidized linoleic acids detected in beef tallow. Significant differences are shown as follows. ^a^ indicates compounds below the lower limit of quantification. * *p* < 0.05 (Student’s *t*-test; Marble 9S-HODE vs. Marble 13S-HODE), ** *p* < 0.01 (Student’s *t*-test; Muscle 9S-HODE vs. Muscle 13S-HODE).

## Data Availability

Not applicable.

## Supplementary Materials

The following supporting information can be downloaded at: https://www.mdpi.com/article/10.3390/metabo12040332/s1. Figure S1: Top 10 metabolites in beef tallow prepared from muscle. Figure S2: Composition of saturated aliphatic acids volatilized by dynamic headspace. Figure S3: Composition of saturated aldehydes volatilized by dynamic headspace. SI Reference [30] were cited in Supplementary Materials.

## Abbreviations

LC-MS/MS	liquid chromatography coupled with a triple quadrupole mass spectrometer system
9-HpODE	9-hydroperoxy-11,12-octadecadienoic acid
13-HpODE	13-hydroperoxy-(9Z,11E)-octadecadienoic acid
9-KODE	9-oxo-octadecadienoic acid
13-KODE	13-oxo-octadecadienoic acid
9S-HODE	9- hydroxyoctadecadienoic acid
13S-HODE	13-hydroxyoctadecadienoic acid
9(10)-EpOME	9,10-epoxy-12-octadecenoic acid
12(13)-EpOME	12,13-epoxy-9-octadecenoic acid
9(10)-diHOME	9,10-dihydroxyoctadec-12-enoic acid
12(13)-diHOME	12,13-dihydroxyoctadec-9-enoic acid
ALOX15	arachidonate 15-lipoxygenase
ALOX15B	arachidonate 15-lipoxygenase type B
COX1	cyclooxygenases 1
COX2	cyclooxygenases 2
ALOX5	arachidonate 5-lipoxygenase
