# Supplementary material for: Production of Hydroxy Fatty Acids, Precursors of γ-Hexalactone, Contributes to the Characteristic Sweet Aroma of Beef
Source: Metabolites. 2022 Apr 6;12(4):332. doi: 10.3390/metabo12040332 (PMC9028887; doi:10.3390/metabo12040332)

Figure S1. Top 10 metabolites in beef tallow prepared from muscle tissue.
The metabolites were quantified using gas chromatography-mass spectrometry.
The water-soluble fraction was extracted from beef tallow and subjected to GC/MS analysis (GCMS-QP2010 Ultra, Shimadzu Corporation, Kyoto, Japan) using conventional methods [1]. Quantification using the peak area ratio (peak height ratio) of the internal standard (5 μg sinapic acid). Data represent the mean ± Standard deviation (n = 3).

No.	Metabolites	Concentration (μg/mL)	Standard deviation
1	Glycerol	39.8	± 3.11
2	Pyrophosphoric acid	19.3	± 1.84
3	Lactic acid	16.2	± 1.31
4	Oxalic acid	11.8	± 1.33
5	Inositol	9.4	± 0.88
6	Glycine	9.2	± 0.66
7	Urea	7.5	± 0.50
8	Pyroglutamic acid	3.6	± 0.42
9	Alanine	3.4	± 0.33
10	Glutamic acid	3.1	± 0.51

1. Ueda, S.; Iwamoto, E.; Kato, Y.; Shinohara, M.; Shirai, Y.; Yamanoue, M. Comparative metabolomics of Japanese Black cattle beef and other meats using gas chromatography-mass spectrometry. *Biosci Biotechnol Biochem* **2019**, *83*, 137-147, doi:10.1080/09168451.2018.1528139.

Figure S2. Composition of saturated aliphatic acids volatilized by dynamic headspace. The composition of saturated aliphatic acids was detected using a gas chromatograph-mass spectrometer. Beef tallow was heated in dynamic headspace and analyzed by thermal desorption unit-gas chromatography-mass spectrometry using an Agilent 7890B gas chromatograph-5977B mass spectrometer equipped with a DB-WAX capillary column.

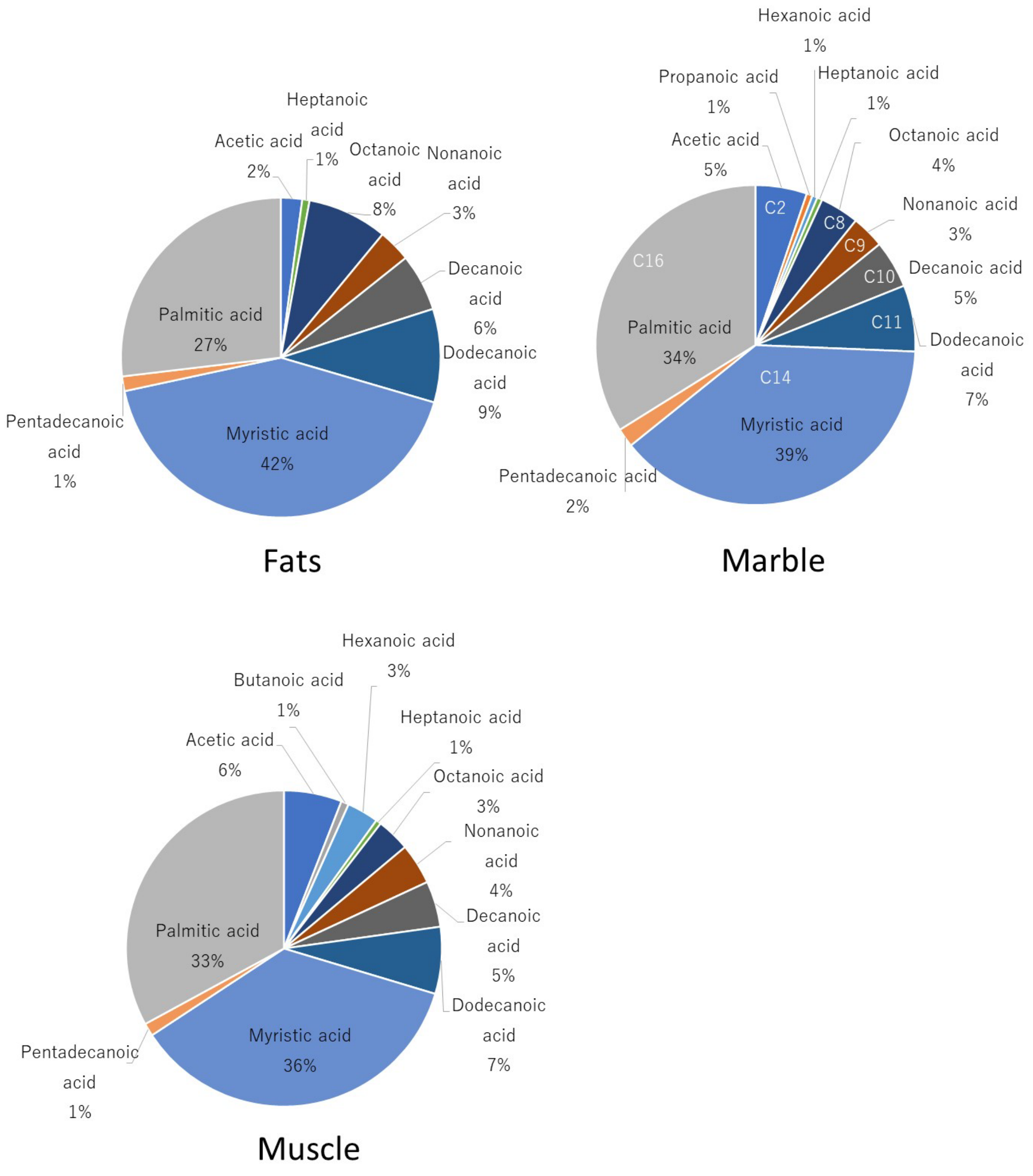

Figure S3. Composition of saturated aldehydes volatilized by dynamic headspace.

The composition of saturated aldehydes was detected using a gas chromatograph-mass spectrometer. Beef tallow was heated in dynamic headspace and analyzed by thermal desorption unit-gas chromatography-mass spectrometry using an Agilent 7890B gas chromatograph-5977B mass spectrometer equipped with a DB-WAX capillary column.

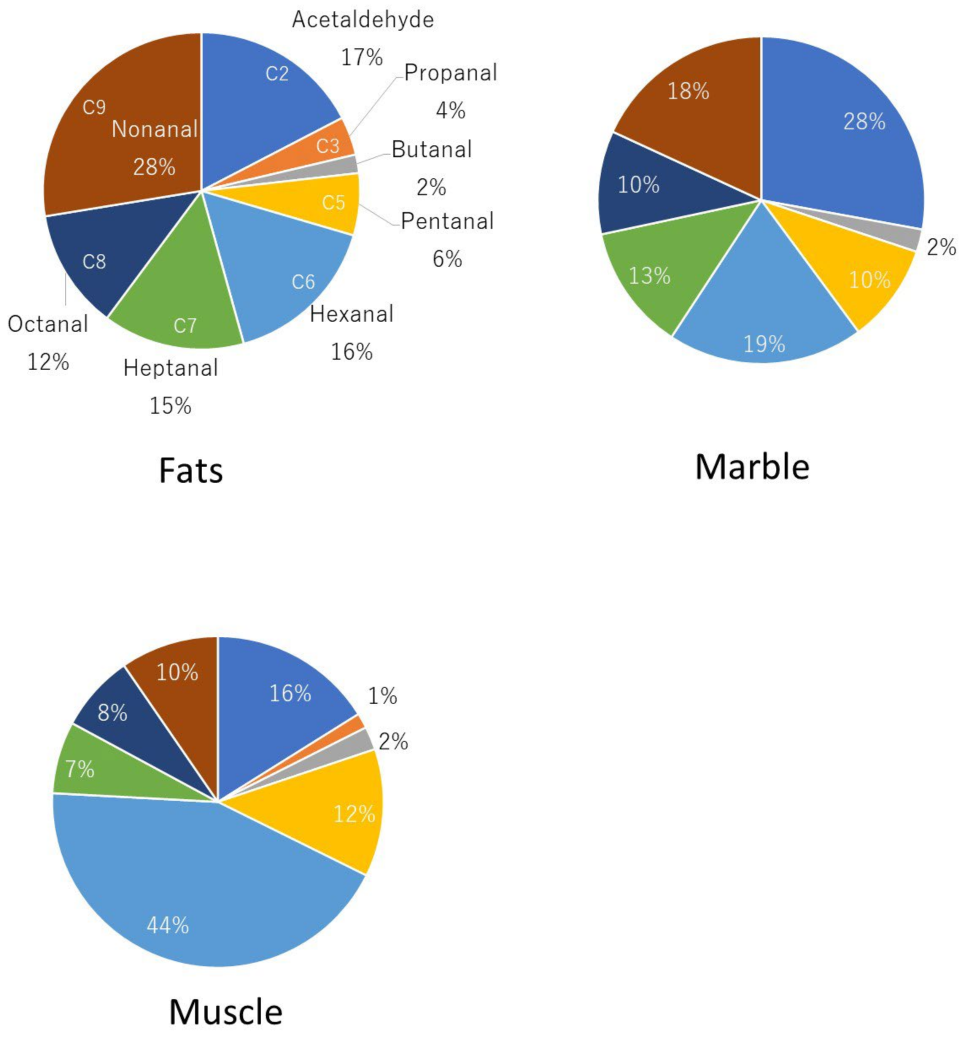

Supplement: Supplementary file 1 [file metabolites-12-00332-s001.zip › metabolites-1655346-supplementary.pdf]
